# Classroom Movement Breaks in Relation to Health and Academic Outcomes: An Umbrella Review

**DOI:** 10.1111/josh.70252

**Published:** 2026-10-07

**Authors:** Nan Dou, Hao Lei, Raquel Arbaiza, Leah E. Chapman, Ann Kuhn, Juliana F. W. Cohen, Erin R. Hager

**Affiliations:** ^1^ Department of Population, Family and Reproductive Health Johns Hopkins Bloomberg School of Public Health Baltimore Maryland USA; ^2^ Department of Epidemiology, Peter O'Donnell Jr. School of Public Health University of Texas Southwestern Medical Center Dallas Texas USA; ^3^ Department of Exercise and Nutrition Sciences University at Buffalo Buffalo New York USA; ^4^ Department of Health Sciences and Nutrition & Center for Health Innovation, Research, and Policy (CHIRP) Merrimack College North Andover Massachusetts USA; ^5^ Department of Nutrition Harvard T.H. Chan School of Public Health Boston Massachusetts USA

**Keywords:** activity disparities, classroom movement break, student well‐being

## Abstract

**Background:**

Classroom movement breaks (CMBs) reduce school‐day sedentary time. This umbrella review synthesized evidence on health and academic benefits of CMBs among school‐aged children and explored outcomes by race, ethnicity, and socioeconomic status (SES).

**Methods:**

A systematic search of systematic reviews and meta‐analyses up to April 2024 was conducted in five databases. Reviews examining CMBs' impacts on health and/or academic outcomes were included. Study quality was assessed using AMSTAR2.

**Findings:**

Sixteen reviews examined PA (physical activity; *n* = 12), classroom behaviors (*n* = 5), academics (*n* = 9, 4‐overall and 6‐domain‐specific academics), cognition (*n* = 8), and health (*n* = 5). All PA‐outcome reviews reported benefits. Most reviews reported improved classroom behaviors (3/5), cognition (6/8), and overall academics (3/4). Findings were mixed for domain‐specific academics and inconclusive for health. Methodological quality of all reviews was rated as low/critically low. Few reviews assessed differences by race, ethnicity, or SES.

**Implications for School Health Policy, Practice, and Equity:**

Although CMBs showed potential benefits, recommendations for school implementation should be made with caution. Equity impacts remain understudied.

**Conclusions:**

CMBs may benefit students' activity, classroom behaviors, overall academics, and cognition, but evidence for subject‐specific academics and health remained mixed or inconclusive. Current findings should be interpreted cautiously. Future research should address low methodological quality, utilize long‐term designs, incorporate uniform CMBs interventions/outcome measures, and address equity gaps.

Adequate physical activity (PA) promotes healthy growth and development among school‐age youth [1, 2]. It is recommended that youth aged 6–17 years engage in ≥ 60 min of moderate‐to‐vigorous PA (MVPA) daily [3]. However, only 20%–28% meet this recommendation [4]. Physical inactivity increases risks of adverse health outcomes, including overweight and obesity, cardiometabolic diseases, and mental disorders [5, 6]. It also negatively impacts students' academic performance through its impact on delayed or impaired cognitive development [7, 8]. Disparities in PA levels are particularly pronounced among youth from urban areas, low‐income households, and ethnic minority groups [9, 10, 11], who are more likely to experience negative health outcomes [12, 13, 14]. These disparities highlight the critical need to explore and implement approaches to equitably provide opportunities for all youth to increase PA and reduce sedentary behaviors.

Youth spend the majority of their waking hours in schools [15]. While schools offer a variety of PA opportunities (e.g., recess, PE classes), students' PA levels remain low. On average, US school‐age youth spend 6–8 h per day sedentary [16], with approximately 63% of school hours spent sitting [17]. Classroom movement breaks (CMBs), involving short bursts of PA performed as a break from academic instruction, have been considered as a sustainable, emerging strategy to counteract in‐school sedentary behaviors [18, 19, 20, 21]. CMBs complement traditional school‐based PA by offering additional, low‐cost, and equipment‐free opportunities for movement during or between instructional hours, making them highly feasible for implementation in diverse school settings. Given their broad reach, researchers have begun examining the impacts of CMBs on child wellness and academic outcomes [22].

Prior systematic reviews and meta‐analyses offer valuable insights into CMB benefits [2, 23, 24]; however, findings were mixed due to variations in study designs and search strategies. First, reviews included studies among youth with broad age ranges, complicating cross‐study comparisons. Second, substantial heterogeneity in study design (intervention vs. observational), CMBs formats (activity types, intensity, and duration), and outcome measures posed challenges to synthesize findings. Third, various terminology use, such as “brain breaks” and “acute breaks,” led to inconsistent identification and inclusion of relevant studies. Lastly, despite extensive research on CMBs and their benefits for school‐aged youth, few studies have reviewed whether CMBs implementation addresses disparities by race, ethnicity, and socioeconomic status (SES).

This umbrella review aims to address these gaps through reassessing findings from systematic reviews and meta‐analyses on the impacts of CMBs in school‐age youth, while evaluating the quality of the included reviews. It also seeks to identify overlooked evidence on differential impacts by race, ethnicity, and SES. Building on Basch's 2011 review of PA and academic achievement [7], this umbrella review provides an updated summary of research findings to inform future school‐based PA practices, particularly CMBs, to support improvements in both health and educational outcomes among students, particularly those facing health and educational disparities.

## 1 Methods

A systematic search of systematic reviews and meta‐analyses published prior to April 2024 was conducted in five databases: PubMed, Embase, Scopus, PsycINFO, and ERIC. Database‐specific indexing terms (e.g., MeSH in PubMed, Thesaurus in PsycINFO) and text words were used to identify relevant reviews (File S1). Specifically, this search focused on school‐based CMB interventions for youth aged 5–18 years old. Observations of school wellness policy implementation specific to CMBs and its related changes in students' outcomes were also of interest. Child outcomes of interest included physical and mental health, health behaviors (e.g., PA levels, sedentary behaviors), cognitive function and development (e.g., attention, concentration), academic performance (e.g., school grades from specific course domains or standardized testing assessments, grade point average, numeracy or language‐related skills), and classroom behaviors (e.g., time‐on‐task (TOT)). The included reviews or meta‐analyses must have involved human participants, been peer‐reviewed, and published in English. Reviews covering a broader age range were included if results specific to youth aged 5–18 could be extracted or if age was analyzed as a moderator. Reviews focusing solely on total, chronic, or acute exercise, and exercise outside classroom or school settings were excluded, unless findings related to CMBs could be separately extracted.

Two reviewers (ND/HL) independently screened titles, abstracts, and full texts for eligibility. Data extraction and quality assessment were conducted in pairs (ND/HL; ND/RA), with each reviewer independently completing their tasks. Final results were compared, and discrepancies were resolved through discussion until consensus was achieved. If a review included studies unrelated to CMBs, only the number of studies specifically related to CMBs was documented when separate findings were reported.

This study used AMSTAR2 (A MeaSurement Tool to Assess Systematic Reviews) to qualitatively evaluate the quality of the included systematic reviews and meta‐analyses [25]. This 16‐item tool evaluates seven critical and nine noncritical domains that impact validity of a review study (File S2). The overall confidence rating for each review was determined as: critically low‐ ≥one critical weakness (with or without noncritical weaknesses); low‐ one critical weakness (with or without noncritical weaknesses); moderate‐ ≥one noncritical weakness; and high‐ no or only one noncritical weakness [25].

This umbrella review has been registered with the international Prospective Register of Systematic Reviews (PROSPERO 2025 CRD420251061959).

## 2 Findings

### 2.1 Study Selection

The literature search yielded 3590 publications from five databases: PubMed (*n* = 1153), Embase (*n* = 403), Scopus (*n* = 1230), PsycINFO (*n* = 451), and ERIC (*n* = 353). A total of 979 duplicates were removed. The title and abstract screening removed 2541 publications, leaving 70 publications for full‐text eligibility review. Fifty‐four publications were further removed according to predefined inclusion and exclusion criteria, leaving a total of 16 systematic reviews and meta‐analyses for final qualitative synthesis. The PRISMA flow diagram (Figure 1) details the publication selection process and exclusion reasons.

**FIGURE 1:**
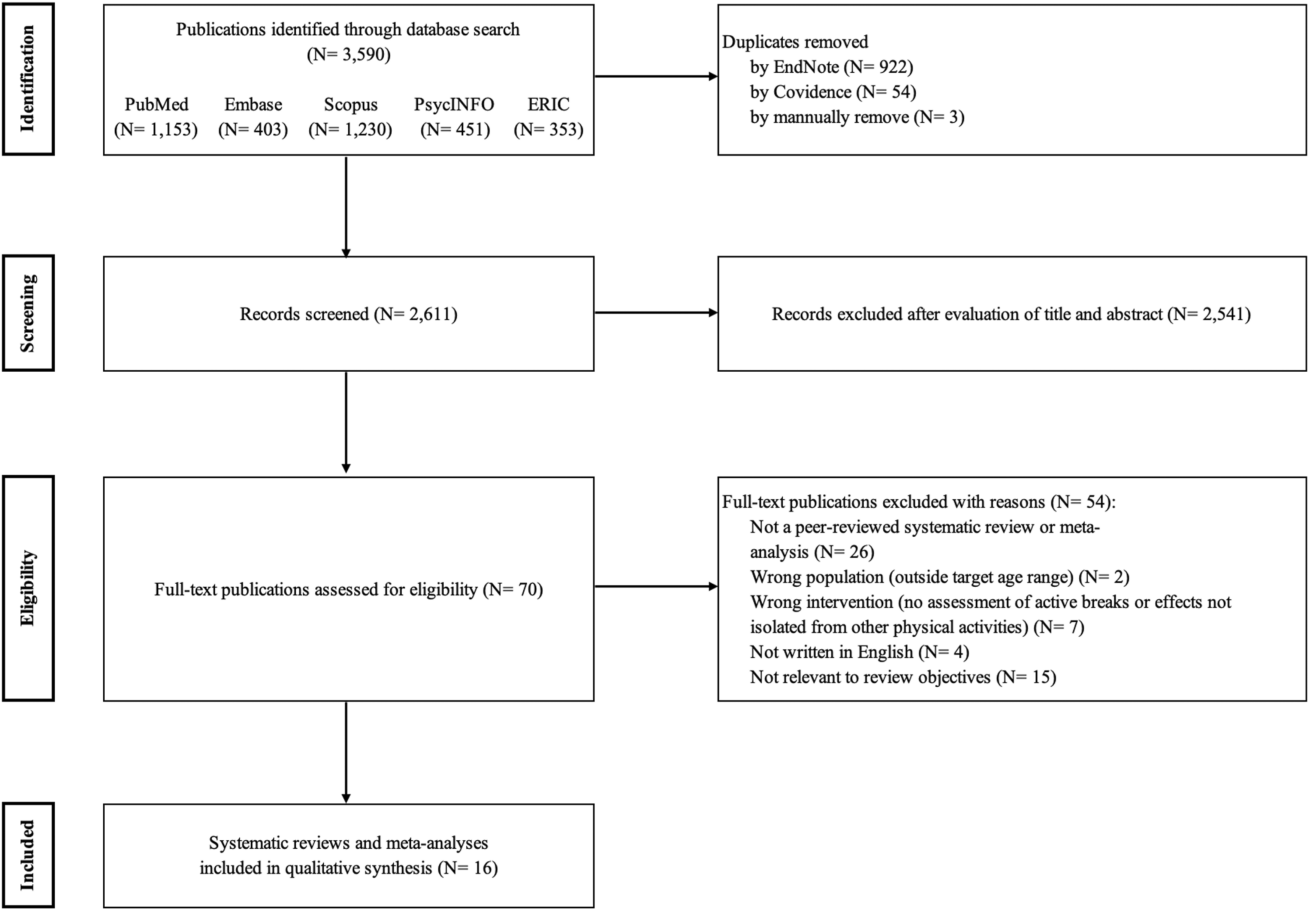
PRISMA flow diagram for umbrella review.

### 2.2 Study Characteristics

This umbrella review included 16 systematic reviews [26, 27, 28, 29, 30, 31, 32, 33, 34, 35] (*n* = 10) and meta‐analyses [36, 37, 38, 39, 40, 41] (*n* = 6) published between 2011 and 2023, each comprising 1–41 individual studies (Table 1). Most reviews included intervention studies [26, 27, 30, 31, 32, 34, 36, 37, 38, 40, 41] (*n* = 11), while a few incorporated interventions, observational studies, prior review articles, or a combination of these [28, 29, 33, 35, 39].

**TABLE 1:** Characteristics and study details of included systematic reviews and/or meta‐analyses.

Authors, year	# of included studies	Study design of included articles	Child age in years	Interventions/exposures	Outcomes				
					Physicalactivity	Health	Classroom behavior	Academic outcomes	Cognition
Systematic reviews									
Barr‐Anderson et al. [27] 2011	28	Intervention	Elementary/primary schools (*n* = 25), junior high (*n* = 1), preschool (*n* = 1)	Short bouts of activity	X	X	X	X	
Donnelly et al. [28] 2016	2	Observational and Intervention	5–13	Classroom physical activity breaks				X	
Daly‐Smith et al. [32] 2018	8	Intervention	8–14	Classroom movement breaks	X		X	X	X
Woods et al. [29] 2021	3	Observational, Intervention, and Reviews	4–19	Physical activity breaks in non‐Physical Education curriculum	X				
Martinez‐Lopez et al. [26] 2021	10	Intervention	6–12	Active lesson breaks				X	X
Pastor‐Vicedo et al. [33] 2021	19	Intervention and Systematic reviews	7–12	School‐based active breaks	X			X	X
Ruhland et al. [31] 2021	20	Intervention	7–16	Classroom‐based physical activity					X
Masini et al. [30] 2022	1	Intervention	16	Active breaks	X	X	X		
Papadopoulos et al. [34] 2022	15	Intervention	6–12	Classroom‐based physical activity	X	X			
Petrigna et al. [35] 2022	41	Observational and Intervention	3.8–11.9	Physically active breaks outdoor and inside of the classroom	X	X	X	X	X
Meta‐analyses									
Masini et al. [39] 2020	22	Observational and Intervention	6–12	Active breaks	X		X	X	X
Infantes‐Paniagua et al. [41] 2021	16	Intervention	6–13	Active breaks					X
Amor‐Barbosa et al. [36] 2022	4	Intervention	6–12	Prolonged sitting time interruption with classroom‐based physical activity	X				
Peiris et al. [38] 2022	9	Intervention	6–13	Short bouts of physical activity inside the classroom during regular class time	X	X		X	X
Carrasco‐Uribarren et al. [37] 2023	8	Intervention	6–12	Prolonged sitting time interruption at school	X				
Zhao et al. [40] 2023	9	Intervention	“Primary school students,” mean age of oldest participants = 11.18 and 11.71 years	Brain Breaks, a classroom‐based physical activity via physical activity‐related videos students can follow	X			X	

Fourteen reviews focused on youth aged 3.8–19 years [26, 28, 29, 30, 31, 32, 33, 34, 35, 36, 37, 38, 39, 41]. The remaining 2 did not specify age ranges but referred to study populations as primary school students or included studies conducted in elementary/primary and junior high school settings [27, 40]. Two reviews included children < 5 years old [29, 35]. In the review by Petrigna et al. [35], 2 of the 41 included studies enrolled children aged 3.8 years, while the remaining studies involved children aged ≥ 5 years. Woods et al. [29] included 3 studies on CMBs, in which the participants were over elementary‐school age. Therefore, this study presented the age group based on the range specified in their inclusion criteria. The description of CMBs varied widely across reviews, with interventions referred to as short bouts of activity [27, 38], classroom PA breaks [28], CMBs [32], active lesson breaks [26], active breaks [30, 33, 39, 41], classroom‐based PA [31, 34], physically active breaks [29, 35], prolonged sitting time interruption [36, 37], and brain breaks [40]. These reviews assessed a range of outcomes. While the majority of reviews evaluated the impacts of CMBs on PA [27, 29, 30, 32, 33, 34, 35, 36, 37, 38, 39, 40] (*n* = 12), fewer examined health outcomes [27, 30, 34, 35, 38] (*n* = 5) and classroom behaviors [27, 30, 32, 35, 39] (*n* = 5). More than half of the included reviews addressed academic performance [26, 27, 28, 32, 33, 35, 38, 39, 40] (*n* = 9) and half addressed cognitive function [26, 30, 31, 32, 33, 35, 38, 41] (*n* = 8).

### 2.3 CMBs Interventions

Across the 16 reviews, CMBs interventions varied widely in format, delivery, intensity, and duration. CMBs were non‐curricular, involving short bouts of PA delivered between or within academic instructions, to interrupt prolonged sitting hours [36, 37]. These PA activities were simple, with minimal equipment required, and adaptable to classroom settings. Some common formats of PA practiced during CMBs included basic, whole‐body movements such as walking or running in place, jumping, squats, push‐ups, sit‐ups, and stretching or relaxation exercises [32, 35]. Several interventions also incorporated structured aerobic activities performed at moderate‐to‐vigorous intensity levels [30, 41], as well as guided activities such as dance, yoga, and Qigong sessions [34]. These interventions were teacher‐led, involved research‐focused protocols, or guided by pre‐designed curriculum, videos, or other commercially available resources. Twelve out of 16 reviews reported on the intensity of CMBs, with most interventions lasting between 2 and 30 min per school day [26, 27, 30, 31, 32, 34, 35, 36, 37, 38, 39, 40, 41]. One review documented sessions extending up to 60 min [37]. Among them, 9 reviews reported the frequency of CMBs, which ranged from once daily to multiple sessions per day or several times per week [26, 30, 34, 35, 36, 37, 38, 39, 40]. Eleven out of 16 reviews reported the duration of CMB interventions [26, 27, 30, 31, 34, 35, 36, 37, 38, 40, 41]. Most interventions were implemented over a few months to one school year [26, 30, 35, 38, 40, 41]. Notably, 4 reviews included long‐term interventions lasting up to 3 years [31, 34, 36, 37], with 1 review reporting an intervention extending to 6 years [27]. The average duration across studies included in this review was approximately 13 months [27]. While prior reviews revealed variations in CMBs interventions, they also demonstrated the wide use of CMBs in school settings across all school‐age youth. Tables 2 and 3 include a detailed description of CMBs interventions.

**TABLE 2:** Summary of findings from 10 systematic reviews examining the benefits of classroom movement breaks on child outcomes.

Authors, year	Interventions/exposures	Outcomes				
		Physical activity	Health	Classroom behavior	Academic outcomes	Cognition
Barr‐Anderson et al. 2011	*Interventions:* Short bouts of activity *Intensity:* 10–15 min on average *Duration:* 2 weeks to 6 years, mean of 13 months.	**↑** *Engagement in fitness skills* **↑** *Physical Activity attitudes* **↑** *Physical Activity level*	**↑** *Fitness measures* Inconclusive/Null findings: Blood pressure BMI Bone health Lean mass	**↑** *Concentration* **↑** *Attentive behavior* **↑** *Time‐on‐task behavior*	**↑** *Academic achievement* Inconclusive/Null findings: District work/social skills progress report Knowledge Missouri academic performance scores	
Donnelly et al. 2016	*Interventions:* Classroom Physical Activity breaks *Intensity:* Not summarized *Duration:* Not summarized				Inconclusive/Null findings: Test Scores: Mathematics, language, reading	
Daly‐Smith et al. 2018	*Interventions:* Classroom movement breaks *Intensity:* 4–20 min *Duration:* Not summarized	**↑** *Physical Activity level, MVPA*		Inconclusive/Null findings: Time On/off task	Inconclusive/Null findings: Test Score: Mathematics	**↑** *Attention* **↑** *Reaction time* Inconclusive/Null findings: Executive function, inhibition, working memory Memory, word recall Processing speed
Woods et al. 2021	*Interventions:* Physical activity breaks in non‐Physical Education curriculum *Intensity:* Not summarized *Duration:* Not summarized	**↑** *Physical Activity level* Inconclusive/Null findings: Cardiovascular fitness				
Martinez‐Lopez et al. 2021	*Interventions:* Short breaks between or within academic lessons. *Intensity*: 4–20 min, daily *Duration:* 3 weeks to up to 1 year				Inconclusive/Null findings: Academic performance	**↑** *Selective attention* Inconclusive/Null findings: Attention Executive function, working memory, inhibition, fluid intelligence Motor function Processing speed
Pastor‐Vicedo et al. 2021	*Interventions:* School‐based active breaks interventions *Intensity:* Not summarized *Duration:* Not summarized	**↑** *Physical Activity level*			**↑** *Academic performance*	**↑** *Attention* Inconclusive/Null findings: Executive function, working memory, inhibition
Ruhland et al. 2021	*Interventions:* Physical activity during regular class time such as short units of physical activity during breaks from lessons *Intensity:* not summarized *Duration:* single day to 3 school years					Inconclusive/Null findings: Attention On‐task behavior
Masini et al. 2022	*Interventions:* Active breaks, high‐intensity interval training (HIIT) *Intensity:* 14 min, 2/week *Duration:* 12 months	**↑** *Physical Activity active engagement* **↑** *Vitality*	Inconclusive/Null findings: Cardiovascular fitness	**↑** *Time‐on‐task behavior* **↑** *Off‐task passive behavior*		
Papadopoulos et al. 2022	*Interventions:* classroom‐based activities *Intensity:* 2–10 min, 1–30 times/week *Duration:* 4 months to 3 years	**↑** *Enjoyment of Physical Activity*	Inconclusive/Null findings: Subjective wellbeing (i.e., Self‐efficacy, Quality of life, Self‐confidence) Other wellbeing and/or enjoyment (i.e., positive mental health, mood enhancement, happiness, and self‐esteem)			
Petrigna et al. 2022	*Interventions:* Physically active breaks outdoor and inside of the classroom *Intensity:* 4–30 min, mean duration of 9 min; five times per week *Duration:* 1 week to 1 school year, mean duration of 13 weeks	**↑** *Physical Activity knowledge* **↑** *Physical Activity level*	**↑** *Decreased body fat* **↑** *Cardiorespiratory endurance* **↑** *General health*	**↑** *On‐task behaviors* **↑** *Overall classroom behavior*	**↑** *Academic performance*	**↑** *Attention* **↑** *Executive function* **↑** *Motor skills* **↑** *Overall cognitive function*

**TABLE 3:** Summary of findings from six meta‐analyses examining the benefits of classroom movement breaks on child outcomes.

Authors, year	Interventions/exposures	Outcomes				
		Physical activity	Health	Classroom behavior	Academic outcomes	Cognition
Masini et al. 2020	*Interventions:* Active breaks *Intensity:* 3–5 min to a maximum of 20 min, 10 min per week to 30 min per day *Duration:* Not summarized	**↑** *Physical Activity level, Step Count* Inconclusive/Null findings: MVPA		Inconclusive/Null findings: Time‐on‐Task	Inconclusive/Null findings: Test Scores: Mathematics, Reading (NA) *NA indicates no meta‐analysis was performed for this outcome	Inconclusive/Null findings: Attention Executive Functions, working memory (NA)
Infantes‐Paniagua et al. 2021	*Interventions:* Active breaks *Intensity:* 4–25 min, 2–5 times per week *Duration:* 2–10 weeks					**↑** *Accuracy (NA)* **↑** *Concentration* **↑** *Selective attention* **↑** *Sustained attention (NA)* Inconclusive/Null findings: Attention, overall attention Inhibitory control Shifting
Amor‐Barbosa et al. 2022	*Interventions:* Prolonged sitting time interruption with classroom‐based physical activity *Intensity:* 5–30 min, 1–3 times per day up to three times per week *Duration:* 5 days to 3 years	**↑** *Physical Activity level, MVPA* Inconclusive/Null findings: Reduce Sedentary Behavior				
Peiris et al. 2022	*Interventions:* Short bouts of physical activity inside the classroom during regular class time *Intensity:* Activity break, 5 min per session per day; In‐classroom physical activity breaks (≥ 10 min), 15–20 min per day *Duration:* 10–4800 min	**↑** *Physical Activity level, MVPA* Inconclusive/Null findings: Reduce Sedentary Behavior Step Count	Inconclusive/Null findings: Aerobic fitness (NA) BMI (NA) Skinfold measurement changes (NA) Test Anxiety		**↑** *Foreign Language Learning* **↑** *Spelling* Inconclusive/Null findings: Test Scores: Mathematics, Reading	**↑** *Attention* Inconclusive/Null findings: Executive function, inhibition, shifting, updating
Carrasco‐Uribarren et al. 2023	*Interventions:* Prolonged sitting time interruption at school *Intensity:* 5–60 min; twice a week to 3 times per day *Duration:* 5 days to 3 years	**↑** *In‐school Physical Activity level, MVPA* **↑** *Reduced in‐school Sedentary Behavior*				
Zhao, et al. 2023	*Interventions:* Brain Breaks, a classroom‐based physical activity program *Intensity:* 3–5 min or 5–10 min per school day *Duration:* 3–4 months, with a minimum of 6 weeks	**↑** *Physical Activity Attitudes* Inconclusive/Null findings: Change in Physical Activity level (NA) Physical Fitness Tests			Inconclusive/Null findings: On‐task Behavior (NA) Short‐term Memory (NA)	

### 2.4 CMBs and PA

Child PA outcomes were evaluated in 12 of the included reviews. Among these, 10 assessed changes in PA levels [27, 29, 32, 33, 35, 36, 37, 38, 39, 40], 3 examined PA‐related knowledge and attitudes [27, 35, 40], and 3 focused on students' active engagement and enjoyment of PA [27, 30, 34]. Additionally, 2 reviews incorporated physical/cardiovascular fitness assessments [29, 40]. Overall, all 12 reviews consistently reported positive impacts of CMBs on PA‐related outcomes, with the exception of physical/cardiovascular fitness, where evidence was limited [29, 40]. Meta‐analyses confirmed significant improvements in step counts [39], overall and in‐school PA and MVPA levels [36, 37, 38], PA attitudes [40], and reductions in in‐school sedentary behavior [37]. One study included in the review by Masini et al. [39] reported sustained benefits of CMB interventions, noting that improvements in students' step counts were maintained at the 3‐month follow‐up [42].

### 2.5 CMBs and Health Outcomes

Child health outcomes were less frequently examined in the identified reviews. Four systematic reviews [27, 30, 34, 35] and 1 meta‐analysis [38] examined child health outcomes, consisting of both physical and psychosocial well‐being. Two systematic reviews [27, 35] and 1 meta‐analysis [38] assessed body composition, physiology, and bone health outcomes. Two reviews supported positive impacts of CMBs [27, 35]. Barr‐Anderson et al. reported mixed findings, with short bouts of activity (10–15 min) linked to improvements in BMI, lean mass, blood pressure, and bone mass; however, results varied by child age, gender, and intervention intensity [27]. Similarly, Petrigna et al. found CMBs (average 9 min/session, five times/week) improved general health and reduced body fat, though subgroup analyses were not conducted [35]. Conversely, the meta‐analysis by Peiris et al. found that implementing short bouts of PA during regular classroom time was not significantly associated with changes in anthropometric measures, such as BMI or skinfold thickness [38]. Three systematic reviews assessed physical or cardiovascular fitness [27, 30, 35], each citing only 1 relevant study, with 2 reporting improvements in muscular strength [27] and cardiovascular endurance [35]. Psychosocial well‐being was evaluated in 1 systematic review [34] and 1 meta‐analysis [38]. Papadopoulos et al. found mixed evidence that only 4 of 13 studies showed significant improvements in subjective well‐being [34]. Peiris et al. conducted a meta‐analysis of 2 studies (*n* = 68) examining test‐anxiety, showing a moderate‐to‐large effect size favoring the CMBs intervention, though results were not statistically significant [38]. While findings were mixed, the evidence suggests that CMBs may support improvements in physical health and fitness, though benefits to psychosocial well‐being remain inconclusive.

### 2.6 CMBs and Classroom Behaviors

The impacts of CMBs on child classroom behaviors were assessed in 4 systematic reviews [27, 30, 32, 35] and 1 meta‐analysis [39]. TOT was the most assessed outcome in all reviews, along with measures of concentration [27], inattentive behaviors [27], and general classroom behaviors [35]. Three out of 4 systematic reviews reported favorable impacts of CMBs [27, 30, 35], whereas the meta‐analysis [39] yielded nonsignificant findings. Three reviews specifically examined how CMBs characteristics influenced these outcomes. Improvements were observed following 4‐min vigorous PA and 10‐min MVPA sessions [32]. Both moderate‐to‐vigorous‐ and light‐to‐moderate‐intensity CMBs were more effective than standard lessons in enhancing classroom behavior [39]. Additionally, long‐term benefits on TOT behaviors were identified [27]. Improvements in attention [27] and overall classroom behavior [35] were consistently observed across studies.

### 2.7 CMBs and Academic Outcomes

A total of 9 reviews, including 6 systematic reviews [26, 27, 28, 32, 33, 35] and 3 meta‐analyses [38, 39, 40], examined the impacts of CMBs on academic outcomes. These reviews assessed a range of academic indicators, including overall academic performance and subject‐specific achievements, such as standardized test scores in mathematics, reading, spelling, and language learning. One review also considered cognitive and behavioral proxies (e.g., short‐term memory and TOT) as academic outcomes [40]. Overall, findings from the included reviews demonstrated positive impacts on overall academic performance, although results were mixed across specific subject domains. Among 4 systematic reviews examining overall academic performance [26, 27, 33, 35], 3 found positive associations with CMBs [27, 33, 35]. Martinez‐Lopez et al. noted that short‐ and long‐term light‐to‐moderate‐intensity CMBs improved academic performance, whereas moderate‐intensity CMBs over 16 months showed no significant impact [26]. Gender differences were also observed, with girls demonstrating greater improvements in mathematics than boys [26]. Reviews examining the impacts on specific subject domains remained mixed. Four reviews assessed standardized subject‐specific test scores [28, 32, 38, 39], with 1 conducting a meta‐analysis [38]. While findings on mathematics and reading were inconclusive, meta‐analytic results showed significant improvements in spelling and foreign language learning [38]. One review examined short‐term memory and TOT as proxies for academic functioning but did not summarize findings due to the limited number of eligible studies [40].

### 2.8 CMBs and Cognitive Function

Cognitive outcomes were assessed across 5 systematic reviews [26, 31, 32, 33, 35] and 3 meta‐analyses [38, 39, 41], although 1 meta‐analysis did not conduct a formal statistical analysis [39]. These outcomes were categorized into key cognitive domains, including attention [26, 31, 32, 33, 35], executive function (e.g., inhibition, updating, shifting) [26, 32, 33, 35, 38, 39], information processing (e.g., reaction time, processing speed) [26, 32], learning and memory (e.g., word recall) [26, 32], motor function and skills [26, 35], and overall cognitive performance [35]. Overall, 6 reviews reported improvements in at least 1 cognitive domain following CMBs interventions [26, 32, 33, 35, 38, 41]. Attention‐related outcomes were examined in 6 out of 8 reviews, with positive impacts found on selective attention, sustained attention, concentration, accuracy, reaction time, and overall attentional performance [26, 32, 33, 35, 38, 41]. Meta‐analyses revealed a moderate effect of CMBs on selective attention [41], a small effect on concentration [41], and a range of small to large effects on overall attention performance [38]. Notably, the magnitude and consistency of impacts varied by intervention intensity, duration, and participants' gender differences [26]. Findings on executive function were inconclusive. Only Petrigna et al. reported a positive impact of a 7‐month PA curriculum on executive function [35]. The meta‐analysis by Peiris et al. also showed no significant impact of CMBs on inhibition, updating, or shifting, with high heterogeneity among studies [38]. Other cognitive outcomes such as reaction time [32], motor function [35], and overall cognitive performance [35] were each reported in a single review, with observed benefits among school‐age youth.

### 2.9 Differential Impacts by Race, Ethnicity, and SES

Only 2 included reviews reported study findings by race, ethnicity, or SES. Infantes‐Paniagua et al. highlighted a single study [43] that reported greater benefits on selective attention following acute CMBs among students from lower‐income families versus those from higher‐income families [41]. Peiris et al. noted that none of the studies included in their review analyzed impacts of CMBs by students' ethnic groups, although 1 study [44] was conducted among African American students [38]. Additionally, 2 reviews acknowledged limitations due to not accounting for SES measures [27, 28].

### 2.10 Methodological Quality Assessment of Included Reviews

All included articles were rated as having either low or critically low methodological quality. Seven [26, 27, 29, 31, 33, 34, 35] systematic reviews and 4 [36, 37, 39, 40] meta‐analyses were rated as critically low quality. A common flaw across nearly all reviews was the failure to provide a list of excluded studies with justifications (*n* = 15), a critical domain in AMSTAR2. Additionally, 10 reviews did not consider risk of bias in individual studies when interpreting or discussing their findings, another critical domain in methodological quality. Among the meta‐analyses, over half (*n* = 4) did not assess publication bias or address its potential impacts. Five reviews were rated as low quality, primarily due to noncritical weaknesses such as missing or insufficient information on a detailed PICO (Population, Intervention, Comparator Group, and Outcome) framework, funding sources for included studies, or discussion of heterogeneity. Overall, the included reviews each missed 2 to 9 critical or noncritical items based on the AMSTAR2 criteria (Table 4).

**TABLE 4:** Methodological quality assessment of included systematic reviews and meta‐analyses using AMSTAR2.

Authors	1	2*	3	4*	5	6	7*	8	9*	10	11*	12	13*	14	15*	16	Number of “NO” of the critical* and noncritical domains	Overall quality
Systematic reviews																		
Barr‐Anderson et al.	N	N	Y	PY	Y	N	N	Y	N/N	N	NM/NM	NM	N	N	NM	Y	4 + 4	Critically low
Donnelly et al.	N	PY	Y	PY	Y	Y	N	Y	Y/Y	N	NM/NM	NM	Y	N	NM	N	1 + 4	Low
Daly‐Smith et al.	N	PY	Y	PY	Y	Y	N	Y	Y/Y	N	NM/NM	NM	Y	N	NM	Y	1 + 3	Low
Woods et al.	N	PY	Y	PY	Y	Y	N	N	Y/Y	N	NM/NM	NM	N	Y	NM	Y	2 + 3	Critically low
Martinez‐Lopez et al.	N	PY	Y	PY	Y	N	N	Y	N/N	N	NM/NM	NM	N	N	NM	Y	3 + 4	Critically low
Pastor‐Vicedo et al.	Y	N	Y	PY	N	N	N	N	N/N	N	NM/NM	NM	N	N	NM	Y	4 + 5	Critically low
Ruhland et al.	N	PY	Y	N	N	N	N	N	Y/Y	N	NM/NM	NM	N	Y	NM	Y	3 + 5	Critically low
Masini et al. (2022)	Y	PY	Y	PY	Y	Y	N	Y	Y/Y	N	NM/NM	NM	Y	Y	NM	Y	1 + 1	Low
Papadopoulos et al.	N	PY	Y	PY	Y	Y	N	PY	Y/Y	N	NM/NM	NM	N	N	NM	Y	2 + 3	Critically low
Petrigna et al.	Y	PY	Y	PY	Y	N	N	PY	N/N	N	NM/NM	NM	N	N	NM	Y	3 + 3	Critically low
Meta‐analyses																		
Masini et al. (2020)	Y	Y	Y	PY	Y	Y	N	Y	Y/Includes only RCTs	N	Y/NM	Y	Y	Y	N	N	2 + 1	Critically low
Infantes‐Paniagua et al.	Y	Y	Y	PY	Y	Y	Y	Y	Y/Y	N	Y/N	Y	Y	Y	Y	Y	1 + 1	Low
Amor‐Barbosa et al.	Y	Y	Y	PY	Y	N	N	Y	PY/N	N	Y/N	N	N	Y	N	Y	5 + 3	Critically low
Peiris et al.	Y	Y	Y	PY	Y	Y	N	PY	Y/Includes only RCTs	N	Y	N	Y	N	Y	Y	1 + 3	Low
Carrasco‐Uribarren et al.	Y	Y	Y	PY	Y	N	N	Y	PY/N	N	Y/N	N	N	Y	N	Y	5 + 3	Critically low
Zhao et al.	Y	Y	Y	PY	Y	N	N	Y	PY/PY	N	Y/N	N	N	Y	N	N	3 + 4	Critically low

*Note:* * indicates the critical items in the AMSTAR2 instrument.

## 3 Discussion

CMBs may benefit students' PA, classroom behaviors, overall academic performance, and cognition. However, evidence for subject‐specific academics and health remained mixed or inconclusive. Limited evidence was found on differential impacts of CMBs in reducing health or academic disparities. The included reviews offered valuable insights into CMBs benefits among school‐age youth, while also highlighting critical gaps in evaluating CMBs' reach and effectiveness among youth from diverse demographic and socioeconomic backgrounds. Caution is needed when interpreting the results of this umbrella review due to the overall critically low to low methodological quality of the included reviews, along with substantial heterogeneity in CMBs interventions and measured outcomes.

The included reviews support potential positive impacts of CMBs on PA outcomes in schools. Earlier recommendations from the Community Preventive Services Task Force suggested that CMBs lasting between 4–10 min, implemented 1–3 times per school day, are effective in increasing PA levels among primary school students [45]. It is also recommended that CMBs delivered by trained classroom teachers significantly enhance students' PA engagement [45]. Although existing reviews suggest that CMBs may improve PA, substantial methodological concerns remain. Future systematic reviews and meta‐analyses should follow established methodological standards to strengthen the evidence base informing the continued implementation and expansion of CMBs in schools.

Conversely, the evidence supporting health outcomes, a logical extension of improvements in PA, was inconclusive. This may be due to the heterogeneity in the format, intensity, and duration of CMBs which may influence their impacts on child health. The majority of included reviews comprised studies implementing CMBs as 5–10 min sessions of MVPA [22]. Among the included reviews, Petrigna et al. reported the most promising physical health benefits of CMBs, including reductions in body fat and improvements in cardiorespiratory endurance and general health, following the implementation of CMBs sessions lasting 4–30 min (average 9 min), five times per week over an average duration of 13 weeks [35]. In other reviews reporting inconclusive findings, CMBs that were not implemented on a daily basis [30] or were of insufficient duration [38], potentially limited the CMBs' effectiveness. Furthermore, none of the included reviews evaluated the sustained impacts of CMB interventions using longitudinal follow‐up designs, while longer follow‐up periods would allow sufficient time to monitor changes in health outcomes. Further research with longitudinal follow‐up after CMBs intervention is warranted to determine the most effective types of CMBs for consistently enhancing child health outcomes. Additionally, further research is also needed to assess impacts of CMBs on the psychosocial well‐being of youth, an area currently underexplored in the literature.

Evidence from this umbrella review suggests that CMBs may positively impact classroom behavior, attention, and academic performance. However, evidence remains inconclusive regarding the “best” type of CMBs for improving these academic‐related outcomes. For example, a meta‐analysis involving 3544 adolescents found that shorter‐duration acute PA interventions more effectively enhance attention and cognitive flexibility compared to longer sessions [46]. Conversely, a recent review suggested that longer (30‐min) CMBs produced the largest effect sizes on attentional outcomes among high school students [47]. Similarly, Masini et al. (2022) identified a study highlighting the benefits of high‐intensity interval training during CMBs on TOT performance [30], supporting prior evidence that high‐intensity exercise may enhance cognitive function in children [48, 49]. Besides, substantial heterogeneity in measurement approaches in academic and cognitive outcomes further limited comparability across studies. For example, academic performance was commonly assessed using standardized test scores across subjects and general academic achievement, though limited details were provided. Cognitive function was evaluated across a wide range of domains, most frequently attention and executive function, with less emphasis on processing speed, memory, and motor skills. Notably, cognitive functioning assessment tools varied by age group and the cognitive domain being measured. However, the included reviews lacked detailed descriptions of the individual cognitive tests used to measure these domains, and nearly no composite score was provided for overall cognitive function. Overall, the heterogeneity in outcome measures made it difficult to conclusively identify the impacts of CMBs on most outcomes related to cognitive functioning. Unlike the meta‐analyses examining other academic outcomes such as overall academic performance and cognitive function, no significant association was found between CMBs and classroom behaviors. This may be attributed to the limitations in the number of included studies [39]. Similarly, reviews examining outcomes beyond PA also showed methodological concerns.

Differential impacts of CMBs on child outcomes by student race, ethnicity, and SES were rarely addressed in prior reviews. More recent literature, not summarized in the included reviews, found that adding daily CMBs in schools would reduce ethnic differences in academic achievement, specifically in math and reading performance, among students who were White, African American, or Hispanic [50]. Given that CMBs are a low‐cost strategy that may potentially increase PA and address classroom behaviors, cognitive function, and academic performance (as shown in this umbrella review), evaluating the impacts of implementation of CMBs in reducing health and academic disparities is a key area of future research.

This umbrella review provides a comprehensive understanding of CMBs implementation and potential benefits for school‐age youth. It has strengths in its standardized search methodology, focused population (school‐age youth), and systematically unifying themes across reviews. It highlighted critical research gaps, particularly the lack of evidence on disparities by race, ethnicity, and SES. Heterogeneity in outcome measures and inconsistent CMBs implementation across studies limited comparability, leaving quantitative analyses of CMBs' impacts on child outcomes not possible. The methodological quality of the included reviews was rated as critically low to low, mostly caused by limited adherence to established methodological standards for systematic reviews and meta‐analyses, including explaining the rationale for article exclusion, and insufficient reporting and interpretation of essential details, including risk of bias. Such methodological weaknesses may compromise the reliability of the synthesized evidence; however, it does not mean that the extracted data are not valid. The widely used AMSTAR2 strictly aligned with established standards for systematic reviews and meta‐analyses, resulting in conservative ratings [51, 52]. Future studies should consider whether additional justification or careful refinement of the rating criteria is needed. Despite weaknesses, this umbrella review offers guidance for future intervention programs with an emphasis on the need for standardized CMBs implementation and outcome assessments across students and settings. It also highlights the need for future research to rigorously evaluate the impacts of CMBs using standardized intervention components, such as activity type, intensity, and duration, while addressing research gaps in understanding disparities across race, ethnicity, and SES. Future review studies should adhere to established methodological standards for systematic reviews and meta‐analyses to generate quality evidence.

### 3.1 Implications for School Health Policy, Practice, and Equity

While the existing evidence is limited by the low methodological quality of included review studies, based on AMSTAR2, given the potential benefits of CMBs including improving PA, classroom behavior, overall academic performance, and cognitive outcomes related to attention, CMBs may still be considered a worthwhile strategy to support the growth and development of school‐age youth. Since CMBs are generally simple, low‐cost, and easy to deliver, schools may consider implementing CMBs without waiting for higher‐quality evidence. However, implementation should be paired with close monitoring and evaluation to support students while also generating practice‐based evidence to inform standardized CMBs protocols and outcome assessment. Although current evidence on equity‐related impacts is limited, existing findings underscore the need for future research to explore disparities by race, ethnicity, and SES. CMBs are typically feasible for broad implementation, particularly in under‐resourced schools.

There are major barriers identified from prior practices, including time constraints, curriculum demands, and difficulties in transitioning back to school work following the movement breaks [53]. Other concerns included lack of teacher confidence in leading activities, limited student participation, and insufficient space in classrooms [42, 54, 55]. For future implementation, schools should address these barriers by providing training for teachers and staff to effectively lead structured movement sessions, allocating sufficient time for CMBs while balancing instructional requirements and PA opportunities, developing strategic plans that specify the appropriate types, intensities, and durations of activities, and providing adequate physical space for safe and engaging participation. At the policy level, clear guidance is needed regarding protected instructional time for CMBs, training resources for teachers and staff, and standardized implementation protocols. To maximize their potential benefits, school programs and policies should prioritize CMBs as sustainable and inclusive practices across diverse educational settings.

## 4 Conclusions

CMBs may benefit students' PA, classroom behavior, overall academic performance, and cognitive outcomes related to attention, with possible benefits for domain‐specific academic performance. Impacts on health outcomes remained inconclusive. These results were derived from selected reviews with low or critically low methodological quality ratings, according to AMSTAR2; therefore, they should be interpreted with caution. Although CMBs vary in format, intensity, and duration, they have been widely implemented in schools. However, limited evidence exists on how these interventions impact children across diverse racial, ethnic, and socioeconomic groups. Future research should prioritize higher‐quality evidence syntheses using standardized review protocols and long‐term evaluations using standardized CMB interventions and validated outcome measures. Priorities should also be placed on developing sustainable school‐level strategies to maximize the reach and equity of CMBs implementation among all students across all school settings.

## Ethics Statement

Preparation of this paper did not involve primary research or data collection involving human subjects, and therefore, no institutional review board examination or approval was required.

## Conflicts of Interest

The authors declare no conflicts of interest.

## Supporting information


**File S1:** Search term used in the umbrella review.
**File S2:** The AMSTAR 2 instrument.

## Data Availability

Data sharing not applicable to this article as no datasets were generated or analysed during the current study.
