# Supplementary material for: Classroom Movement Breaks in Relation to Health and Academic Outcomes: An Umbrella Review
Source: J Sch Health. 2026 Oct 7;96(10):e70252. doi: 10.1111/josh.70252 (PMC13644376; doi:10.1111/josh.70252)
Supplement: Supplementary file 1 [file JOSH-96-0-s001.docx]

**Supplemental File 1. Search Term Used in the Umbrella Review**

**PubMed:**

school [Mesh] OR school [tiab] OR schools [tiab] OR classroom [tiab] OR classrooms [tiab] OR classroom-based [tiab] OR class [tiab] OR class-based [tiab]

AND

(movement[tw] AND break[tw]) OR "movement breaks" [tw] OR "exercise break" [tw] OR "exercise breaks"[tw] OR (active[tw] AND learning[tw] AND break*[tw]) OR (active[tw] AND lesson*[tw] AND break*[tw]) OR "active break*"[tw] OR "physical break*"[tw] OR "movement learning" [tw] OR (physical[tw] AND activit*[tw] AND break*[tw]) OR (physical[tw] AND exercise*[tw] AND break*[tw]) OR (acute[tw] AND exercise*[tw] AND break*[tw])

OR

"movement break" [tiab:~1] OR "movement breaks" [tiab:~1] OR "exercise break" [tiab:~1] OR "exercise breaks"[tiab:~1] OR "active learning break"[tiab:~1] OR "active learning breaks"[tiab:~1] OR "active lesson break"[tiab:~1] OR "active lesson breaks"[tiab:~1] OR "active lessons break"[tiab:~1] OR "active lessons breaks"[tiab:~1] OR "active break"[tiab:~1] OR "active breaks"[tiab:~1] OR "physical break"[tiab:~1] OR "physical breaks"[tiab:~1] OR "movement learning" [tiab:~1] OR "physical activity break"[tiab:~1] OR "physical activity breaks"[tiab:~1] OR "physical activities break"[tiab:~1] OR "physical activities breaks"[tiab:~1] OR "physical exercise break"[tiab:~1] OR "physical exercise breaks"[tiab:~1] OR "physical exercises break"[tiab:~1] OR "physical exercises breaks"[tiab:~1] OR "acute exercise break"[tiab:~1] OR "acute exercise breaks"[tiab:~1] OR "acute exercises break"[tiab:~1] OR "acute exercises breaks"[tiab:~1]

Note: the exercise terms have been intentionally repeated with proximity operators to capture a small additional number of articles.

**Embase:**

(movement NEAR/1 break*) OR (exercise NEAR/1 break*) OR (active NEAR/3 break*) OR (physical NEAR/3 break*) OR (movement NEAR/1 learning) OR (acute NEAR/1 exercise* NEAR/1 break*)

AND

'primary school'/exp OR 'middle school'/exp OR 'high school'/exp OR 'school'/de OR 'high school student'/exp OR 'middle school student'/exp OR school:ti,ab,kw OR schools:ti,ab,kw OR classroom:ti,ab,kw OR classrooms:ti,ab,kw OR 'classroom based':ti,ab,kw OR class:ti,ab,kw OR classes:ti,ab,kw OR 'class based':ti,ab,kw OR student*:ti,ab,kw

**Scopus:**

school OR schools OR classroom OR classrooms OR classroom-based OR class OR class-based OR student*

AND

(movement W/1 break*) OR (exercise W/1 break*) OR (active W/3 break*) OR (physical W/3 break*) OR (movement W/1 learning) OR (acute W/1 exercise* W/1 break*)

*Formatted to paste into Advanced Search:*

( TITLE-ABS-KEY ( ( movement W/1 break* ) OR ( exercise W/1 break* ) OR ( active W/3 break* ) OR ( physical W/3 break* ) OR ( movement W/1 learning ) OR ( acute W/1 exercise* W/1 break* ) ) ) AND ( TITLE-ABS-KEY ( school OR schools OR classroom OR classrooms OR classroom-based OR class OR class-based OR student* ) )

**PsycINFO:**

school OR schools OR classroom OR classrooms OR classroom-based OR class OR classes OR class-based OR DE "Primary School Students" OR DE "Elementary Schools" OR DE "High School Students" OR DE "Elementary School Students" OR DE "Schools" OR DE "High Schools" OR DE "Junior High Schools" OR DE "Middle Schools"

AND

(movement N1 break*) OR (exercise N1 break*) OR (active N3 break*) OR (physical N3 break*) OR (movement N1 learning) OR (acute N1 exercise* N1 break*)

**ERIC:**

(DE "Secondary School Students" OR DE "High School Students" OR DE "Junior High School Students" OR DE "High School Freshmen" OR DE "High School Seniors") OR (DE "Middle School Students") OR school OR schools OR classroom OR classrooms OR classroom-based OR class OR classes OR class-based OR DE "Schools" OR DE "Elementary Schools" OR DE "Middle Schools" OR DE "Secondary Schools" OR DE "High Schools" OR DE "Junior High Schools"

AND

(movement N1 break*) OR (exercise N1 break*) OR (active N3 break*) OR (physical N3 break*) OR (movement N1 learning) OR (acute N1 exercise* N1 break*)

**Supplemental File 2. The AMSTAR 2 Instrument**
